# Korean Immigrant Mothers and the Journey to Autism Diagnosis and Services for Their Child in the United States

**DOI:** 10.1007/s10803-023-06145-w

**Published:** 2023-10-24

**Authors:** Hyeyoung Kim, Sohyun An Kim, Han Lee, Robin Dodds

**Affiliations:** 1 Human Development & Psychology, University of California Los Angeles, Los Angeles, CA 90095 USA; 2 Center for Dyslexia, Diverse Learners, and Social Justice, University of California Los Angeles, Los Angeles, CA 90095 USA; 3 Division of Special Education & Counseling, California State University, Los Angeles, CA 90032 USA

**Keywords:** Autism identification, Korean immigrant mothers, Qualitative research, Family support

## Abstract

Since autism diagnosis is directly linked to the availability of supportive services, identifying best practices for early diagnosis of autism has long been a concern of professionals and families. Meanwhile, studies show persistent racial disparities in autism diagnosis. Although numerous clinical diagnostic guidelines have been published, there is not enough discussion of diagnostic procedures through the lens of culturally diverse families. *Purpose.* This study focuses on the autism diagnostic experiences that Korean immigrant mothers had with their children. *Methods.* Eleven first-generation Korean-American mothers of children with autism were included in the study. The data was collected using semi-structured interviews in Korean. *Results.* The main five factors (i.e., cultural beliefs and values, language barriers, complex emotions, immigration and navigating systems, and facilitators and assets) that mainly influence the diagnosis process were identified through thematic analysis. *Conclusion.* Dynamics are interactive within and between the factors, influencing the entire diagnostic process by either delaying or facilitating the identification of a child’s autism and the provision of treatment.

**Supplementary Information:**

The online version contains supplementary material available at 10.1007/s10803-023-06145-w.

Autism is a life-long neurodevelopmental condition characterized by challenges with social communication, restricted interests, and repetitive behaviors. Early signs of autism can be observed during the toddler and preschool years (American Psychiatric Association, 2013). Research has shown that early identification of autism plays a critical role in fostering long-term positive outcomes due to the malleability of the brain during this early period of development (e.g., Boyd et al., 2010). While research suggests that autism can be reliably diagnosed as early as 14–24 months (Ozonoff et al., 2015; Pierce et al., 2019), according to the US Center for Disease Control and Prevention (CDC), approximately 58% of children on the autism spectrum did not have a comprehensive evaluation on record until after their third birthday, although developmental concerns were shared with professionals before 36 months for 85% of the children (CDC, 2018). Research also shows that there are persistent racial disparities in receiving a timely diagnosis (CDC, 2020; Mandell et al., 2009; Wiggins et al., 2020), suggesting that some children from ethnic minority groups may not receive the early intervention services they need in order to improve long-term outcomes (Harris et al., 2014).

While there is a growing body of literature on the experiences of culturally and linguistically diverse (CLD) parents with their autistic child, little is known about such experiences for Korean American families. Asian Americans were the fastest-growing cultural group in the United States between 2000 and 2010, and a 36% increase in the Korean population was observed during this period (Hoeffel et al., 2012). Although the Asian population comprises many distinct nationalities with their own unique characteristics (e.g., marital contexts, religion, language), researchers tend to consider it as a homogenous group. Therefore, although Asian American students may be overrepresented in the category of autism in special education in the US (Marks & Kurth, 2013), cultural variables that may impact the autism identification process and navigating the system have not been adequately scrutinized across subgroups (e.g., Korean, Chinese, Filipino).

Research has examined contributing factors to challenges with the diagnostic process and accessing services for CLD families overall, highlighting the unique challenges arising from cultural beliefs and variables. Cultural mismatches in the perceptions and knowledge surrounding autism were mostly found to serve as a barrier (Blacher et al., 2014). Many CLD parents reported misconceptions of and unfamiliarity with autism prior to their child’s diagnosis in the US (Papoudi et al., 2021). Examples of misconceived causes of their child’s autism held by CLD parents included issues with diet or stress levels during pregnancy (e.g., Ijalba, 2016; Gilligan, 2013), microwaving baby food, vaccines, and overuse of technology (Wang & West, 2016). Consequently, the child’s disability is attributed to parental carelessness and unhealthy habits, which may cause shame and delay the diagnostic process.

Systemic barriers to accessing timely and equitable evaluations and services, as well as systemic cultural biases, have been reported by racially and ethnically diverse parents (CDC, 2020; Wiggins et al., 2020). For instance, parents from culturally diverse families such as Black, Latinx, and Korean stated that their concerns about their children’s behavioral or developmental issues were dismissed or minimized by the possible cultural bias of service providers, resulting in delays in diagnosis (Stahmer et al., 2019). It was noted that there is limited information available for the identification of children with autism in non-Western settings, where there is relatively less awareness and fewer supportive services for affected children (see Barbaro & Halder, 2016 for a review). Since diagnostic decisions on autism can easily be influenced by cultural variables related to social communication (e.g., eye contact), etiological factors (e.g., maternal place of birth), or a combination of both (Samadi & McConkey, 2011), such a lack of consistency in diagnostic procedures across cultures may impede US providers to honor an autism diagnosis previously issued from a non-Western country.

Across cultures, parents of children with autism experience a range of emotions, including shock, despair, and sadness (see Makino et al., 2021 for a review), which impact family dynamics and stress levels. The cultural beliefs of Asian immigrant parents that stem from collectivism may generate emotional dependence on people outside their immediate family. However, Korean immigrant mothers may lack familial support due to being isolated from their extended family in their origin country (Khanlou et al., 2017). Further, since individuals from collectivistic societies understand their personal identity in the context of their place within the group (Darwish & Huber, 2003), there was a pronounced tendency toward self-sacrifice by parents from Asian cultures in order to bring forth hope. For example, many Asian immigrant mothers perceive their obligations to include giving up their personal goals in order to provide care for their child with autism (Wang & West, 2016; You & Rosenkoetter, 2014). These types of changes in family dynamics often resulted in increased conflicts between fathers and mothers in the family unit (Kim & Kim, 2017), while their shared immigration status helped strengthen bonds within their family unit for some (You & Rosenkoetter, 2014; Zechella & Raval, 2016).

Since the autism diagnosis is directly linked to the availability of supportive services, identifying best practices for diagnosing autism during early childhood has long been a concern of professionals in the field. Meanwhile, studies have shown that the prevalence of autism diagnoses is different for different racial and ethnic groups (Becerra et al., 2014). Aspects of diagnosing autism, especially for those from culturally and linguistically diverse family backgrounds, have proven challenging for professionals as they require additional considerations (i.e., culture and language) for assessment (Aylward et al., 2021). Although numerous clinical diagnostic guidelines have been published, there is not enough discussion of culturally diverse families’ experiences of diagnostic procedures and access to services post-immigration (Sritharan & Koola, 2018). Understanding families’ diagnostic experiences with autism will be an important starting point in optimizing care for children with autism and their families.

The purpose of this study is to identify unique factors that Korean immigrant mothers face as they address the needs of their children and navigate through the various support and services by answering the following research questions:

How do Korean immigrant mothers experience the diagnostic process for their children with autism in the United States?

How do Korean immigrant mothers experience post-diagnostic systems (e.g., education, social services, healthcare) for their child with autism in the United States?

The study utilized a secondary data analysis adapting interview data obtained from Kim and Dodds (in press)’s original study, *Disclosing the Child’s Autism Spectrum Disorder: Perspectives of First-Generation Immigrant Korean Mothers*. The original study, which employed semi-structured interviews, explored participants’ perception of and willingness to disclose the autism diagnosis of their children to others (i.e., friends, family members, and community stakeholders). In regards to disclosing a child’s disability, healthcare providers are often the first to reveal a child’s autism diagnosis and to refer parents to medical, therapeutic, and educational services. Thus, the original study’s interview protocol included diagnostic and service-related questions (see Appendix 1). Although the current study was conducted in the form of secondary data analysis, the findings were differentiated from Kim and Dodds’ original study (in press) by centering on participants’ experienced challenges in the diagnostic process and obtaining services following it. Furthermore, these findings are significant in that they contribute to literature distinguishing what is common to all families from what may be more unique to CLD families in search of support for their children. While many families experience challenges during the experience of diagnosis, the lack of knowledge about autism, it’s symptoms and etiology; isolation from extended family; cultural beliefs and values; and linguistic barriers likely intensify the emotions around the diagnosis, the impact on family cohesion, and frustrations in acquiring access to services that are aligned with family values.

## Methods

### Participants and Recruitment

The eligibility for participation was as follows: Korean American immigrant mothers older than 18 who confirmed that the child was served under the autism category on his or her IEP and who used Korean as a primary language. A snowball sampling strategy was employed to gather additional interview participants. Specifically, the first author contacted the first three participants from a support group for Korean American mothers who have children with developmental disabilities, where the first author had been involved as a member for six years at the time of the interviews. The initial participants then voluntarily invited other participants using their personal networks. Six out of the 11 total participants had personal connections with the first author. See Table 1 for full participant information. All procedures described were approved by the University Institutional Review Board prior to recruitment and data collection.

**Table 1:** Participants and child characteristics

ID	Yrs in US	Home-language	Child Pseudonym	Primary language of child	Child Age	Age at initial diagnosis	Diagnosis/es	Country of initial diagnosis
P1	13	Korean	Sophia	English	12	5	Autism	US
P2	18	English	Matthew	English	10	4	Autism/ ADHD	US
P3	3	Korean/English	Wallace	English	7	4	Autism	S. Korea
P4	4	Korean	Henry	English	4	2	Autism/ SCD	US
P5	20	Korean	James	English	10	4	Autism	US
P6	13	Korean	Lucas	English	9	3	Autism/ ADHD	US
P7	10	Korean	Joshua	English	11	3	Autism	US
P8	25	Korean/English	Kai	English	9	3	Autism/ ADHD	US
P9	17	English	Caleb	English	10	6	Autism	US
P10	7	Korean	Nathan	English	7	3.5	Autism/ ADHD	US
P11	12	Korean	Brian	English	9	3	Autism	S. Korea

*Note.* Yrs = years. ADHD = Attention Deficit Hyperactivity Disorder. SCD = Social Communication Disorder

### Data Collection

The interviews were conducted as part of a previously completed research study analyzing participants’ perspectives of their experience in the US as Korean immigrant mothers of children with autism spectrum disorders. The process of disclosing their child’s disability to education, healthcare, and community stakeholders by Korean American mothers is presented in Kim and Dodds (in press). Through the initial data analysis for the study, questions regarding the identification process and access to services were included and were reported for this manuscript. Such questions were “When did you first learn about your child’s autism?” “When/how/where did you receive the diagnosis?” “What was your or your family’s reaction to your child’s autism diagnosis?” The overall interview protocol of the original study is presented in Appendix 1. The current study focused on the experiences and challenges faced by mothers when attempting to obtain the appropriate resources for their children with autism while navigating through cultural disparities.

All interviews were conducted over Zoom or phone and ranged in duration from an hour to an hour and a half. The interviews were recorded and encoded in a password-protected folder on the first author’s computer. Each interview was transcribed, and identifiable information was removed. Any available recordings were deleted, and transcriptions saved according to a participant ID number once validated for accuracy. These files were shared among the authors in secure cloud-based folders. Considering the sensitivity of the interview questions, all participants were notified before the start of the interview that they could skip any questions and stop the interview at any point.

It is noted that six out of 11 participants were acquainted with the first author and the other five were recruited through snowball sampling strategy. Despite the possible pressure that could come from the direct/indirect relationship between the first author and the members of the support group, all participants were willing to participate in the interview upon request. Generally, when the interviewer and interviewee’s race/ethnicity and/or cultural backgrounds match, the interviewee’s trust in the interviewer escalates (Sands et al., 2007). However, it might be difficult for both the interviewer and interviewees to maintain an objective distance. Moreover, some participants might have been hesitant to talk about their personal stories, afraid of the potential impact on their future relationships. With this in mind, participants were encouraged to talk about their experience at their preferred comfort level. Lastly, at the end of the interview, all participants were asked if the interview questions had caused them any kind of psychological trauma or emotional turbulence, and all participants answered no to this.

Interviews were conducted in Korean, and all went through a translation process. First, the first author translated all the transcripts from Korean to English and checked each for accuracy. The second and third authors, who have similar linguistic backgrounds, translated five or six transcripts each from Korean to English independently and in parallel. The translators met weekly throughout the process and any discrepancies in translations were discussed and resolved between the first three authors.

### Data Analysis Plan

#### Positionality

All interviews were conducted by the first author, who is a Korean immigrant to the US and the mother of a child with a developmental disability. Not only does her personal background match the inclusion criteria of the participants for this study, but she shares commonalities as a participant in the same support group. Furthermore, the professional experiences of the first author, such as her career in special education in South Korea, provided additional insight into the cultural differences in the education of children with disabilities across both countries. The second, third, and fourth authors share the positionality of teaching experiences with students with disabilities, and the fourth author is also the parent of a child with autism. They individually provided perspectives on the analysis of the interview data as outsiders. The commonalities and discrepancies within the research team contributed to the data analysis as they collectively deciphered cultural values and their impacts on the identification process for the participants’ children.

#### Thematic Analysis

Thematic analysis is a method for systematically identifying patterns or themes in a qualitative dataset (Braun & Clarke, 2006; Maguire & Delahunt, 2017). Thematic analysis is chosen for its potential to provide a richly descriptive account of complex phenomena. Six stages of the thematic data analysis process were used: familiarization with data, generating initial codes, searching for themes, reviewing potential themes, defining and naming themes, and producing the report (Braun & Clarke, 2006).

The interview data were inductively analyzed, relying on transcripts of interviews using Dedoose (2021) software. The first and the second authors played an active role in developing the code book through a regular weekly meeting. They first read the 11 interview transcripts together several times and developed preliminary codes that were relevant to the research aims (i.e., autism diagnosis and services). These codes were the result of observing and discussing repeated patterns of experiences, perceptions, and ideas of the participants. The two authors encountered the following three situations while developing the preliminary codes: (1) they developed the codes collaboratively; (2) one author developed the codes and the other agreed to them; and (3) any discrepancies with the codes were resolved by engaging in open discussions to come to an agreement. While the first and the second author reviewed and generated the official codebook, the third author participated approximately 30% of the time to confirm the relevance of the preliminary codes and coding process. The appropriateness of these codes was discussed and confirmed through biweekly meetings with all four authors.

Table 2 depicts a sample of the process through which each of the preliminary codes underwent in developing the factors mentioned throughout this study. In addressing the research aims of gaining insight into what impacts the journey of autism diagnosis and services for children with autism in Korean immigrant families, five major factors were generated from the themes. The themes were consolidated and labeled as major factors in order to summarize and provide an experiential roadmap of what the participants have faced in their pursuit of advocating for their children’s needs.

**Table 2:** Inductive process of analysis from interviews with Korean Immigrant Mothers

Research Question	What factors impact first-generation Korean-immigrant mothers’ experience during the identification and post-diagnosis process of their children with autism?		
Preliminary Codes	Codes	Themes	Factors
Misinformation: autism can be cured Change in attitude towards disability	1.1 Illness/sickness 1.2 Autism is curable 2.1 Autism as disorder with very severe symptom only	1. Cultural awareness on autism 2. Cultural mismatch in perception of autism between two cultures	Cultural beliefs and values
Clinician’s delivery of information about ASD/ Unclear Confusion due to dual language use	1.1 Difficulty discerning the cause of delay in language development; 1.2 Clinician’s mixed guidance toward bilingualism 2.1 Limitation in accessing information and communicating; 2.2 Ineffective communication with professionals about the diagnosis due to language barriers	1. Child’s dual language use 2. Language barriers	Language barriers and bilingualism
Lack of interest or empathy Progress leading to inflated hopes	1.1 Anxiety/shock/fear; 1.2 Distress triggered by external/internal factors 2.1 Distress due to immigrant status 3.1 Desperate to seek support	1. Emotion associated with diagnosis 2. Emotions associated with immigration 3. Emotions as motivator	Complex Emotions
Delay in accessing services (after diagnosis) Lack of resources	1.1 Discrepancy in the autism diagnostic process between two countries 1.2 Formal diagnosis (reevaluation) needed in the US to access services	1. Barriers in diagnostic process in the US	Immigration and navigating systems
Initiating identification/ Husband’s suggestion Support from others	1.1 Encouragement from family members to obtain autism diagnosis 2.1 Husbands’ (or in-laws’) psychological support	1. Family support 2. Spousal support	Facilitator

## Findings

The purpose of the current study was to identify factors that influence the experiences of Korean immigrant mothers during their child’s autism identification process and obtaining supportive services. Specifically, the unique challenges and difficulties that the mothers experienced as Korean immigrants throughout these processes were explored. Five factors emerged from thematic analysis that impacted the diagnostic process: cultural beliefs and values, language barriers, complex emotions, immigration and navigating systems, and facilitators. Despite some commonalities throughout the diagnostic process, each participant’s journey was uniquely influenced by one or more of the five factors.

### Cultural Beliefs and Values

The participants’ understanding of autism which initially derived from their home country culture could influence the process of autism identification in their children and their preferences for support and services. For example, most participants said that they understood autism to be a severe disorder until their children were diagnosed with it, because in Korea, autism is generally recognized as a very profound disability rather than a spectrum. As a result, some participants and their families paid less attention to, or even overlooked, their child’s early signs of autism, delaying diagnosis. P9 stated, “The first thing I knew about autism was that there were no reactions to other people or anything else. So, people often say it’s like talking to a wall because autistic people never have a reaction, like a wall. That’s why I never thought that my child was autistic.”

Culture also influenced participants’ word choices when discussing their children’s autism. Some participants did not use the word “disability” when discussing their children but rather expressed that their child suffered from a sickness or illness. P7 said that she accepted her son’s disability as though he was “sick” (아프다: ahpeuda). As a result, she determined that taking her son to the hospital in order to treat the “illness” was the correct decision. Similarly, P9 believed in “the development of medicine” and hoped for a “drug to cure this [autism]”. These participants were more likely to view a child’s disability as a curable disease rather than a life-long condition.

The abovementioned limited awareness of and pathological approach to autism may be attributed to Korean society being less inclusive for people with disabilities. Most mothers said that they had limited interactions with people with disabilities, so they had no reason to think about the issue until their children were diagnosed. A few shared that they had never met people with autism growing up. P2 said, “I did not have any opportunities to encounter people with a disability, so I was not interested [in people with disabilities] at all.” P1 pointed out that the Korean public education system failed to provide an inclusive learning environment for children with disabilities. She said that “becoming an adult without having proper access to educational opportunities integrating with people with disabilities results in a lack of awareness of disability.”

### Language Barriers and Bilingualism

Upon receiving an autism diagnosis, a lack of access to culturally and linguistically relevant information was apparent to the participants. The fact that the mothers’ native language was not English appeared to have a negative impact on service satisfaction and inhibited access to information related to intervention services. P3 was frustrated because she thought the language barrier was limiting her access to service information. She added, “American parents probably have more information than I do…I do my best as a foreigner. I write emails and phone calls using my poor English, but I’m not sure if it is good enough”. P2 also mentioned, “…since English is not my first language…I cannot make an argument very well…If I were good at persuading [in English], I might be able to get what I want.”

Since the primary language of all 11 mothers in the study is Korean, their children developed Korean as their mother tongue and began to learn English later at school or when they started receiving disability services. Participant 10 explained how her son’s autism diagnosis was postponed despite her concern about his language delay: “The psychologist said that my son might be displaying speech delays because he was bilingual. So [the psychologist] wasn’t sure if his delay was due to autism or from being bilingual.” Some mothers did not even suspect the child’s language delay as an early sign of a potential disability until the symptoms became more prominent because they assumed they were a natural consequence of being raised as bilingual. P9 said, “I found out about his disability too late. I thought he had a speech delay because he was bilingual”. As such, bilingualism appears to interfere with both parents’ and professionals’ detection of child’s language delay.

Moreover, the interviews indicated that the mothers spearheaded the development of their children’s English rather than Korean after they were diagnosed with autism. On top of the non-linear language development typically seen in bilingual children, the diagnosis of disability motivated mothers to further challenge their children’s language development. Since most support and services for children were conducted in English, the mothers felt the need to provide directions in English when their children exhibited challenging behaviors. P3 explained, “His language development is unique. He is usually most comfortable speaking in Korean. But all he hears is English. For example, the therapist’s instructions are all in English. So, when problem behavior occurs, explaining in English helps him to calm down, but in Korean, it is a little difficult.” P4 decided to stop speaking Korean within the family in order to speed up her son’s English development. She shared, “It took about 6 months to completely extinguish Korean… He was confused and had a hard time. Now we speak English exclusively.”

Many mothers had conversations with their clinicians about bilingualism when their children were diagnosed and received varying instructions and suggestions regarding their children’s language development. Some clinicians recommended enforcing monolingualism with English, because they lived in the US, and others were encouraged to use both English and Korean. Since the mothers recognized their clinicians as experts, they respected and followed their advice. However, it was not known whether the interviewees were aware of whether there was a difference of opinion on bilingualism among clinicians.

### Complex Emotions

Mothers reported experiencing various powerful emotions, such as shock, fear, despair, and depression when their children were diagnosed with autism. Some said that they initially denied their children’s diagnoses, resulting in delays in accessing supportive services. Mothers also experienced anxiety rooted in uncertainties regarding their child’s prognosis and their future. P8 said that she had a hard time accepting her son’s autism because she was worried about adequately caring for him for the rest of her life. P9 said that it felt like she was losing her child, knowing that the future she had imagined could not become a reality. She said, “The hardest thing right now is thinking about whether Caleb and I will ever be able to have an in-depth conversation…it is so heartbreaking to think that the future I imagined for myself and my child is so uncertain now.”

While some emotions concerning their children’s diagnosis naturally arose, in some cases, they were triggered by external factors such as careless and hurtful comments from members of the family and or community about their child’s disability. For instance, P8 reported that her own mother blamed her for the child’s autism by saying that she focused too much on her own career instead of focusing on her pregnancy and the child’s development. In addition, P3 and P11 expressed frustration about how they themselves, and their children, were excluded from social opportunities like birthday parties due to their autism. P3 explained, “If my son had been invited, of course he would have gone. I am a person who attends everywhere. But they excluded us…that did not feel very good.”

Despite facing such social adversities, it was found that the mothers experienced powerful emotions that acted as strong motivators for problem solving and advocacy, despite their language and cultural barriers. P7 said, “It was a difficult time […] and I never had enough hope that it would work…But the person I am now thinks that he has made this much progress because I worked so hard back then.” The more emotional challenges the mothers faced, the more motivated they were to overcome systemic barriers and to advocate for support and services. However, this had an adverse effect on parents where they felt overwhelmed in managing their child’s disability.

Moreover, the emotional difficulties the participants faced were amplified by special circumstances related to their immigrant status, such as separation from family or close friends and their unstable status of residence. P3 and P4’s families were originally planning on living in the US temporarily as expats, but they decided to permanently immigrate in order to utilize autism support and services for their children. Nevertheless, these mothers constantly experienced anxiety due to their immigration status. P4 explained,

Since I don’t have a green card or social security number…I am always anxious on the road because I may end up in a disadvantageous position if an accident were to happen. I am originally a confident and determined person, and it’s not like me to be so anxious.

Similarly, P3 explained that, until her family’s temporary visa status transitions to permanent status in the US, she would always be worried about losing her child’s autism services if they had to move back to Korea. In addition, she expressed her gratitude and remorse towards her husband who sacrificed his career success in Korea to support their child’s access to therapies in the US.

### Immigration and Navigating Systems

Parental confusion resulting from discrepancies in the autism diagnostic systems between Korea and the US were most clearly pronounced in P11’s narrative. Other participants’ experiences aligned with hers and further contributed to this theme.

Several mothers who came to the US with children who had already been diagnosed reported that it was difficult to get a diagnosis at an earlier developmental stage in Korea. In a culture where autism is recognized as a severe disorder, a high diagnostic threshold exists despite detection of early signs before the age of three. For instance, P11’s son was diagnosed with autism in Korea before their family immigrated to the US. The timing of his diagnosis was delayed due to the more rigorous diagnostic criteria in Korea. Therefore, when she detected early signs prior to his 3rd birthday, she had to travel to several hospitals for her son to be diagnosed with autism. She said exasperated,

I didn’t know he had autism, but it was so challenging to care for him (one day) I saw an online forum. I read some postings there and realized that he would actually fall under the autism spectrum disorder, under the US standard…but they [psychologists in Korea] said they don’t give a diagnosis until the child turned three. So, I took him to a rehabilitation center nearby where they had a pediatric department. He got evaluated again there, but they also didn’t give him an autism diagnosis. So, I thought he didn’t have autism.

Participant 10 had a similar experience; “It is harder to obtain an autism diagnosis in Korea. Nathan was sent for evaluation for autism in Korea, but they said his symptoms were not severe enough to receive the diagnosis.”

P11 was eventually able to get her son diagnosed in Korea once he was over the age of three. During this time, P11’s acquaintances, pediatrician, psychologists, preschool teachers, and her parents recommended raising her son in the US for better services and support for autism since her husband was already in a degree program in the US. Typically, mothers and working professionals in Korea perceive the US as an ideal place to raise children with disabilities considering the quality of special education services through the public school system. P3 provided an example of her friend’s son to explain the differences in special education services between Korea and the US. She said,

Her child [in Korea] is in a special education class at his elementary school, and her son was assigned to a military service worker [as a 1:1 aide] …what would a military service worker know? … in the US, my son is in an autism class where there are two assistant teachers and one homeroom teacher. These are people who at least know about our children and what to do when our children have problematic behaviors. But military service workers don’t know anything.

However, it took a considerable amount of time for P11’s son to enter the US public education system and receive private therapeutic services. P11 was unaware of the varying availability of autism services across different regions in the US. She and her family initially settled in a small rural town where the autism service infrastructure was not well-established. She noted,

When I did an online search about autism intervention in the US, I found that some children were receiving 30 h of ABA therapy a week. But it turned out to be all about people in California [not the place where I live]…At the time, the law precluded them from covering ABA services in my state. When we moved here, [my] state law started allowing insurance to cover ABA services. But then there weren’t any ABA therapy centers in the new neighborhood (laugh).

Furthermore, after immigrating to the US, P11’s son was required to undergo another evaluation in order to access private (e.g., ABA) and public (e.g. IEP) special education services. Due to the issues surrounding access to their health insurance and relocating within the US, it was not until her son turned seven that he started receiving adequate services, even though his first diagnosis was given at age three.

In sum, these mothers chose to immigrate to the US with expectations of better quality and access to education and support options for their children. However, those who immigrated without prior knowledge about the systemic challenges (e.g., availability of services, the necessity for re-evaluation) were forced to undergo much trial and error when trying to secure services, which served as a barrier to timely support.

### Facilitators

While Korean immigrant mothers reported challenges such as cultural and linguistic barriers, and emotional hardship, various situations and contexts also served as facilitators for their child’s diagnostic process. Broadly, professionals and family dynamics served as facilitators that expedited the identification process and helped the mothers to accept their child’s autism.

As noted previously, a bilingual home environment may serve as a barrier in the diagnostic process. However, P5 reported that her child’s pediatrician was adamant that her son receive an evaluation for autism by a developmental pediatrician. Due to the pediatrician’s strong recommendation, she was able to prevent further delay of the onset of her son’s autism support and services. In addition, the school system was also found to act as a facilitator for the identification process. For example, P9’s child lives in a trilingual home environment (Korean, Spanish, and English). Her son exhibited a jovial demeanor and maintained good eye contact, so she perceived signs of language delay as a result of exposure to multilingualism. However, when her son transitioned to kindergarten, his teacher recommended that an evaluation was necessary. Similarly, while P1’s daughter was attending preschool, she received intervention services through the school district due to developmental delays, despite not having a formal diagnosis of autism. However, upon transitioning to kindergarten, she received her diagnosis through the school psychologist, which prompted an autism intervention.

Likewise, mothers reported that support from family members can serve as a facilitator in accepting the diagnosis, finding emotional comfort, maintaining a positive attitude, and overall partnership with service providers. Specifically, some mothers acknowledged havinfacilitateg the support of their husbands and their extended family members played a significant role in overcoming obstacles along the way. For instance, when the father was more accepting of the child’s autism diagnosis, this buffered the mother’s emotional distress and relationship with extended family members, thus further positively impacting the intervention plan. P8 explained,

To be honest, I wanted to wait it out a little longer. I hoped he wasn’t autistic. But my husband thought it was better for [the child] to get the diagnosis early and start the support and services right away…My husband thought we should be more hopeful since [the child] is not in any physical pain, and autism can be improved with intervention. Since I was so devastated, he tried to cheer me up this way. He was very supportive.

P3 admitted that her mother-in-law played an important role in helping the entire family to accept her son’s disability.

My mother-in-law said that my child was strange. She started by saying that he couldn’t make eye contact, so we might want to go to the hospital…Although my parents didn’t seem to understand it [the diagnosis] well…My mother-in-law loves my son the way he is. She really loves my child. So, her family accepted it rather easily.

On the other hand, relatively clear gender roles in the traditional family dynamics relating to child-rearing were observed. The mothers were the children’s primary caregivers (including the affected children) and carried out domestic duties. At the same time, the fathers exercised authority in the family by making important decisions and taking full responsibility for the household economy. Therefore, the mother was responsible for the plan for care, support, and services for children with autism, while the father paid more attention to the financial burdens, including the extra expenses associated with the child’s disability-related services (i.e., private therapies), for the family. P4 noted, “My husband is working hard to make money and I do my best to take him to therapies and provide support in ways I learned from his therapists.” These mothers emphasized that such distinct delegation of parental roles worked to their benefit.

## Discussion

The analysis of interview data highlighted culture as a crucial factor in the diagnostic process, and also when accessing services for the participants. Three areas, such as the perception of autism as a severe disorder, word choices reflecting the medical model approach to autism, and insufficient awareness from the parents themselves about autism as a result of limited historical interactions with individuals with disabilities, were found. The findings aligned with previous research and further contributed to the literature by emphasizing the impact of cultural barriers on immigrant families when acquiring access to services to support their children with autism (Stahmer et al., 2019; Fong et al., 2021). More specifically, the combination of the cultural lens through which they viewed what disability entails, and the stigma of being diagnosed with autism in Korea, impacted how the participants acquired support and opportunities to support their child with autism.

When exploring the participants’ perceptions of autism, it was found that their core beliefs and understandings were shaped by the cultural beliefs of their native country. The view of autism as a severe disability influenced the participants to disregard early autism signs and delayed the diagnostic process. This resulted in a more reactive approach to accessing services, rather than taking proactive measures to seek the appropriate support. This is significant to the present research as it contributes to the cruciality of culturally responsive practices within the healthcare system in order to raise awareness in immigrant families regarding areas such as early signs, intervention, and disability itself.

The influence of culture was also evident in their choice of medical terms to discuss their child’s disability. For example, they often described their autistic child as “*ill*” and some admitted that they were waiting for medical technology to advance so that their child’s autism could be cured. Because Korean mothers fear the label of autism as it implies chronicity as opposed to recovery (Grinker & Cho, 2013), it is possible that describing autism as a curable medical condition may temporarily provide the parents with hope, and can potentially motivate them to seek support. However, such an approach can in fact exacerbate the stigma because it focuses on what a person cannot do (Anderson-Chavarria, 2022) and creates a boundary between ‘normalcy’ and ‘abnormalcy’ (Waltz, 2008). Rather, raising awareness on neurodiversity within Korean society and helping them to perceive autism as a part of human diversity can potentially yield long-lasting positive effects on removing stigma around the disability.

Further, lack of interaction, experiences, and inclusion with people with disabilities in Korea, resulted in participants having insufficient knowledge regarding how to interact with and accept this subgroup of individuals. Thus, encouraging society and various communities, systems, and countries to increase avenues of exposure and awareness of disability is essential, as this could potentially result in a decline in the stigmatization of disability as previously indicated (Neville & White, 2011).

The participants also experienced language barriers which contributed to the challenges in accessing resources and services for their children and/or understanding the extent of their child’s needs. More specifically, professionals who provide developmental screening and assessment in languages other than English are extremely limited (Zuckerman et al., 2014), and this creates a roadblock for parent-reported assessments (Stahmer et al., 2019) which are a crucial part of the diagnostic process. Moreover, while family-centered practices were found to be linked to effective parent involvement and successful early intervention services (Mas et al., 2019), linguistic mismatches between parents and professionals may impede the development of authentic partnerships.

Furthermore, participants received inconsistent recommendations regarding the use of their native language versus English. However, regardless of the recommendations received, all participants reported speaking only English in the home. This may be due to the mothers’ desire to help their children develop their language skills rapidly so that they will be functional both in and out of the home. Nevertheless, it has been evident through previous research that for bilingual children, the use of both languages, particularly at an early age, is beneficial to the child’s overall language development and does not hinder the development of one language over the other (Drysdale et al., 2015). As language is undoubtedly one of the most important aspects of family dynamics, and Korean parents tend to respect and abide by the recommendations provided to them by healthcare professionals (An, 2017), it is imperative that professionals present accurate, evidence-based information about maintaining their heritage language while supporting English development for children with autism.

Our findings revealed that the mothers’ strong emotions related to the diagnosis of their children stemmed from both internal turmoil and external social factors. The mothers were especially frustrated with emotions instigated externally (e.g., hurtful comments and blame from others) because they had relatively less control over these triggers. Since family and community members each maintained different perceptions of autism (Grinker & Cho, 2013), feedback and reactions regarding the diagnosis were varied, and differences in perception held by others appeared to instigate actions and remarks that could be taken as hurtful.

On the other hand, internally generated emotions were caused by the mothers’ own perceptions towards autism. Emotions that the participants experienced, such as shock, despair, and depression, were uniquely internalized into a form of motivation that forcibly drove participants to seek support and services that were not readily available due to systemic barriers. Additionally, consistent with the assertions of Kish-Gephart et al. (2009), this motivation also inspired participants to problem-solve and overcome various challenges that prevented access to support. This perspective on motivation aligns with self-efficacy theory (Bandura, 1982) through which participants’ emotions influenced their expectations for success by acting and seeking resources and support to benefit their child’s future. Such problem-focused coping strategies may also facilitate the mothers’ development of positive perceptions associated with post-traumatic growth (Zhang et al., 2015; Dodds & Singer, 2018).

Findings from this study imply that there are several barriers for toddlers with autism receiving a formal diagnosis in Korea. For instance, participants who attempted to pursue evaluation in Korea struggled to receive a diagnosis despite recognizing their child’s early signs of autism. It was reported by participants that the heightened threshold for diagnostic criteria was one of the barriers against timely identification in Korea. Additionally, mothers were told that clinicians were unable to provide a formal diagnosis of autism for children under three. As previous literature suggests that the prevalence of autism in Korea will increase as more accurate diagnoses are made and awareness of the autism spectrum increases (Kim et al., 2011), future studies must examine the extent to which these factors contribute to the discrepancy between the actual prevalence of autism and the identified diagnosis.

The current study revealed that the systemic discrepancy between the diagnostic process in the US and Korea contributes to the delays in accessing appropriate services in the US. More specifically, autism diagnoses received in Korea were not readily recognized in the US for some participants, which contributed to the delayed onset of intervention services. However, there is a lack of recommendations in the current literature to support children with a foreign autism diagnosis in the US healthcare and educational systems. As it is evident that early intervention is effective in fostering long term positive outcomes for autistic individuals, there is a pressing need to determine ways to minimize the gap in accessing support and services incurred by immigration processes.

In Asian culture, the stigma of having a disability is a serious concern within the family (Saetermoe et al., 2001). Despite generational changes and social movements that have steered the culture away from a more patriarchal society, Asian society continues to be embedded with male-dominated practices and perspectives (Liu & Iwamoto, 2006). Similarly in Korean culture, the position of the father’s perspective and attitude is highly valued in the family dynamic. Thus, if the father is more accepting of the child’s disability and plays an active role in the intervention plan of their child, the less likely a mother will have to endure stress related to the care of their child’s needs. The clear delegation of responsibilities between paternal and maternal roles could contribute to more efficient teamwork within a family system. With consideration to further rectify implementation of culturally relevant practices within the education and healthcare system, these findings stress the differences within gender roles and family systems that should be honored and recognized by professionals and organizations when supporting a child’s needs.

### Limitations and Future Directions

This study was conducted through secondary data analysis. Therefore, questions specific to this study might not have been addressed in the original study. The recurrence of the challenges in obtaining and receiving diagnostic support were prevalent in the participants’ responses and was highlighted as part of this study. Second, there were discrepancies between the time the parents were interviewed and the child’s diagnosis (i.e., at least two years, at most eight years). Thus, the interview data relied on the parents’ episodic accounts of their diagnostic experiences. Lastly, the results cannot be generalized across all Korean American immigrant families with autistic children as it only includes a small number of participants. In addition, various family situations during the diagnostic process that are not captured in this study may influence the experience.

It is recommended that future research extends from the current study by exploring various family structures. Specifically, the role of fathers, the influence of their experiences and perspective through their cultural background may provide a more comprehensive understanding of the factors contributing to the delay in diagnostic processes and challenges in navigating the support system. Future studies examining these types of experiences within multi-racial family structures are also suggested. In addition, participants in the current study had high educational attainment. While Esterline and Batalova (2022) indicate that Korean immigrants have higher educational attainment compared to the overall foreign- and native-born population in the US, we caution against the generalization of the current findings to the broader Korean immigrant population.

Nevertheless, the current study has important implications in recognizing the role of collaboration among healthcare, education, and family stakeholders in support of student outcomes. The more aligned these systems of support are in diagnostic practices, cultural awareness, and accessibility of resources, the more likely it is that a Korean American student with autism will benefit across environments. As early intervention is the key to optimal outcomes, any delay in services provided is detrimental to CLD students with autism and their families.

**Fig. 1:**
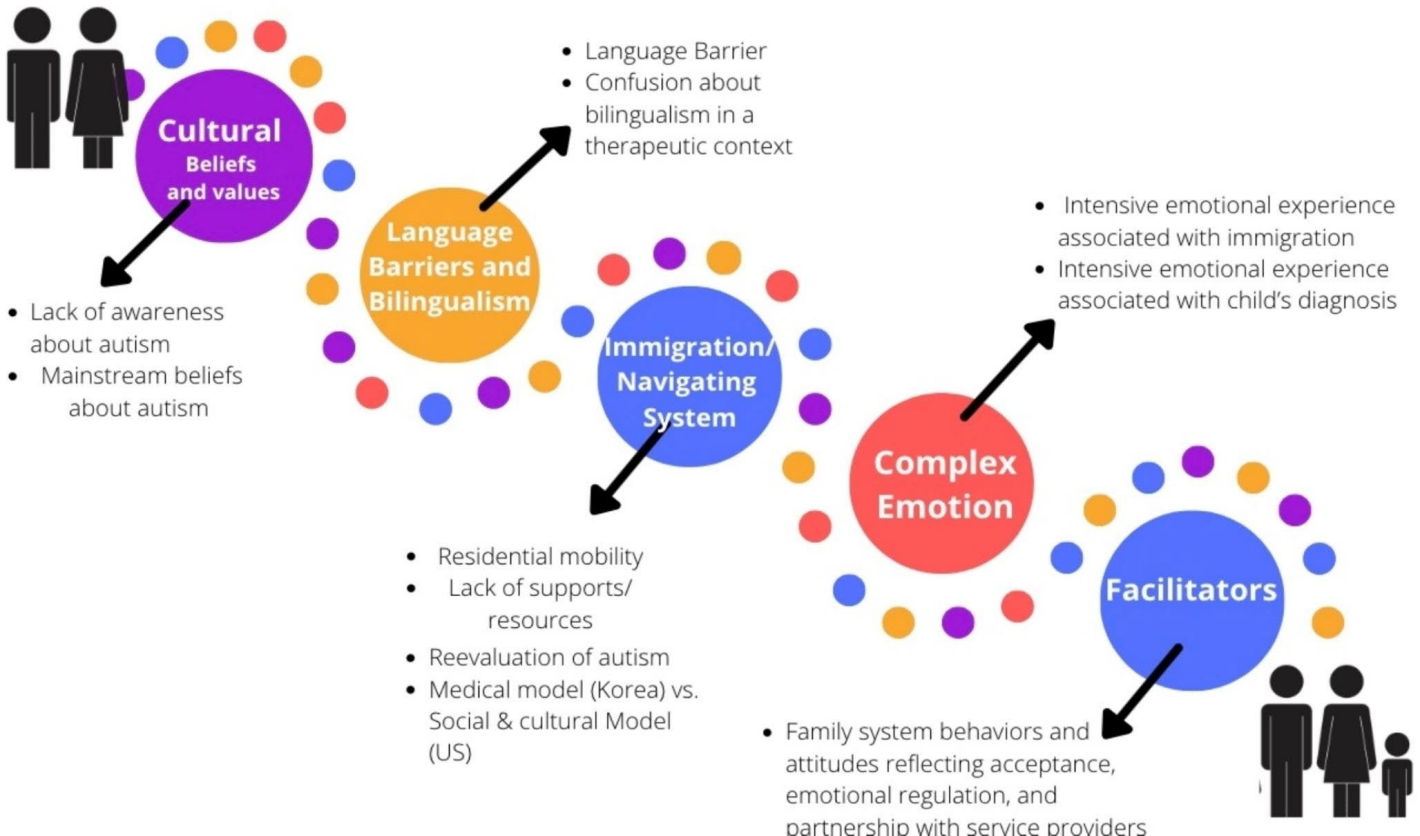
Illustration of the five factors influencing Korean immigrant mothers’ journey in their children’s autism diagnosis

## Electronic supplementary material

Below is the link to the electronic supplementary material.

Supplementary Material 1
