# Supplementary material for: Korean Immigrant Mothers and the Journey to Autism Diagnosis and Services for Their Child in the United States
Source: J Autism Dev Disord. 2023 Oct 24;54(12):4624–36. doi: 10.1007/s10803-023-06145-w (PMC11549211; doi:10.1007/s10803-023-06145-w)
Supplement: Supplementary file 1 — Supplementary Material 1 [file 10803_2023_6145_MOESM1_ESM.docx]

**Appendix 1**

**Interview Protocol on Disclosing the Children’s Disability**

1. Get to know each other briefly
2. Demographic questions
   1. About yourself

Current place of residence/ number of years lived in the US/ age/ gender/ first language/ spoken language at home/ occupation/ highest level of education

- 1. About your family

Relationship to the child on the spectrum/ number of children living in household/ number of children on the spectrum

- 1. About your child on the autism spectrum

Gender/ age of the child/ age of the child when they received the diagnosis/ country the child received the diagnosis/ child’s level of spoken language/ whether receiving IEP at school/ placement at school (full inclusion/ partial inclusion/ specialized school/ private school/ homeschooling/ others)

1. Autism spectrum-related questions
   1. Did you know about autism before your child was diagnosed?
   2. When did you first learn about autism spectrum disorder?
   3. What were your thoughts on autism in the past?
2. Diagnosis-related questions
   1. When did you first learn about your child’s autism?
   2. When/ how/ where did you receive the diagnosis?
   3. How did you decide to receive the diagnosis?

- Can you tell me what happened when you decided to receive the autism diagnosis?
- Who was the first person to notice the child’s autism? And what happened?
- Who was the person encouraging or discouraging you from receiving a diagnosis? And what happened?
  1. Where did you receive your child’s diagnosis?
- In case the child received the autism diagnosis in Korea, 1) Have you received the diagnosis in the US? 2) tell me about the process
  1. What were the challenges of receiving a diagnosis in the US?
  2. What was your or your family’s reaction to your child’s autism diagnosis?
  3. What were the challenges of receiving supportive services?

1. Has your perception of disabilities changed since you came to the U.S.?

a. Do you think there are differences between Korea and the U.S. regarding the perception of disability/autism?

b. Has your perception of disability/autism changed since you came to the U.S.?

c. Does the perception of disability/autism affect daily life?/ how does it affect it?

1. Experience of talking about a child’s autism to others
   1. Tell me about your experiences of talking about autism.
   2. Did you tell your family/ friends/ co-workers/ community members about your child’s autism?
   3. What were the challenges of talking about your child’s autism?
   4. Were there any cultural differences in disclosing your child’s autism (i.e., reaction, acceptance)
2. Experience talking with children about their disability

a. Have you ever discussed with your child about his or her disability?

b. If so, when and how did you approach it?

c. What is the most difficult thing in disclosing disability to your child?

d. How do your language barriers affect talking about?

e. How does the mother’s culture affect talking about your child’s disability to him or her?

1. Perception toward the U.S. special education service

a. In order to receive special education services, you are required to disclose your child’s disability. Did you have any difficulty in this process?

b. Have you ever been concerned that disclosing your child’s disability to the school would have a disadvantage to your child (socially and/ or academically)?

c. Have you ever discussed receiving special education services with your child?
